# The role of 'omics' in the quest to eliminate human filariasis

**DOI:** 10.1371/journal.pntd.0005464

**Published:** 2017-04-20

**Authors:** Sara Lustigman, Alexandra Grote, Elodie Ghedin

**Affiliations:** 1 Molecular Parasitology, New York Blood Center, New York, NY, United States of America; 2 Center for Genomics and Systems Biology, Department of Biology, New York University, New York, NY, United States of America; 3 College of Global Public Health, New York University, New York, NY, United States of America; University of Washington, UNITED STATES

## Current treatment strategies for the filariases

Lymphatic filariasis (LF) is a disease affecting approximately 120 million people in over 73 countries [1] and caused by infection with a group of filarial nematodes transmitted by mosquito vectors. *Wuchereria bancrofti* is responsible for approximately 90% of the disease worldwide, while the remaining cases are due to *Brugia malayi* and *B*. *timori* [2]. These filarial nematodes have important social and economic impact causing high morbidity and serious illnesses resulting in social stigmatization, marginalization, and loss of work for the afflicted [3]. In 2000, The Global Programme to Eliminate Lymphatic Filariasis (GPELF) was launched with the objective to eliminate this disease as a public health problem by 2020 [4]. The eradication of lymphatic filariasis relies on mass drug administration (MDA) using the three drugs currently available for treatment: diethylcarbamazine (DEC), albendazole, and ivermectin. GPELF also has made significant progress in many countries, delivering, between the years 2000 and 2014, 5.6 billion treatments to more than 763 million people living in 61 countries. It was estimated that this directly prevented 36 million clinical cases and saved 175 million disability adjusted life years (DALYs) [5]. However, it is unlikely that LF will be eliminated by the target year of 2020 as 55 of the 73 countries considered to be endemic for LF in 2015 still require MDA [6]. Moreover, GPELF has lagged in Sub-Saharan Africa where only 2 of 35 LF-endemic countries have stopped MDA and started post-MDA surveillance. Notably, recent studies show that single-dose combination therapy with the three antifilarial drugs (ivermectin/albendazole/diethylcarbamazine, or IDA) appears to be superior to current regimens used in the elimination programs, which may help accelerate LF elimination in Africa. Although it has not yet been tested, IDA may also be useful for treating onchocerciasis [7,8]. However, reports of drug resistance to ivermectin and albendazole [9,10] as well as serious concerns about using DEC in Sub-Saharan Africa because of ocular adverse events after DEC treatment of onchocerciasis in the past, makes the discovery of novel drugs against onchocerciasis imperative [11].

The debilitating eye and skin disease known as onchocerciasis is caused by *Onchocerca volvulus*; it is the world's second-leading infectious cause of blindness in humans with 99% of cases in Sub-Saharan Africa alone. Current estimates put 120 million people at risk and 17 million already infected, of which 1.2 million suffer from vision impairment or blindness [12,13]. While the focus of the control efforts has been to alleviate morbidity and lost productivity, onchocerciasis has more recently been targeted for elimination [14,15]. The three past and present onchocerciasis control programs; OCP, the Onchocerciasis Control Programme; APOC, African Programme for Onchocerciasis Control; and OEPA, the Onchocerciasis Elimination Program for the Americas, rely on annual or biannual MDA of ivermectin, a therapy effective at killing microfilariae but not adult worms, with the goal of interrupting disease transmission. However, as the programmatic goals shifted from reducing public health impact to active elimination by 2025, sole reliance on ivermectin is threatened by contraindications in areas co-endemic for loiasis, an inability to break transmission in some foci, and the emergence of drug resistance. Even successes in Latin America and small foci in Africa [16–22] now must be weighed against the fact that since 1995 only a 31% reduction in the incidence of onchocerciasis has been achieved in Africa [13]. APOC in 2015 predicted that 1.15 billion treatments until 2045 will be needed to achieve elimination [23]. Other neglected tropical disease experts doubt that onchocerciasis can ever be eliminated through MDA with ivermectin alone [24] especially given that MDA of ivermectin cannot be used in 11 Central African countries co-endemic with *Loa loa* infections due to the risk of severe adverse events [6,25]. Moreover, many areas of sub-Saharan Africa do not implement onchocerciasis MDA programs in areas of hypoendemicity, which could lead to reintroduction in areas undergoing MDA [26]. Of equal concern is the potential emergence of ivermectin-resistant *O*. *volvulus*, limiting the long-term effectiveness of MDA [27–29] and, in time, undermining gains achieved by onchocerciasis control programs. Complicating the resistance issue is that ivermectin is not administered to children ≤5 years old, and a macrofilaricidal drug, doxycycline, cannot be given to children ≤9 because of the limiting indications for these drugs. These children then are not only vulnerable to infection, they become reservoirs for transmission [30]. For these reasons, in 2014 APOC called for the development and testing of new *O*. *volvulus* technologies, including the development of novel macrofilaricidal drugs, vaccines, and diagnostic biomarkers of infection [31].

## The last decade of ‘omics’ data for filarial worms

In 2007, the same year PLoS NTD was launched, the first parasitic nematode genome was published with the draft genome of *B*. *malayi* [32,33]. In 2009, a review in PLoS NTD focused on helminth genomics and its implications for human health [34], predicting that new sequence information would revise what we knew of the host-parasite, vector-pathogen, and filaria-symbiont relationships. At that time, the genomes of *B*. *malayi* and its endosymbiont, *Wolbachia* (*w*Bm) [35] were available along with expressed sequence tags or EST datasets of other filarial parasites, enabling the construction of a microarray containing 18,104 elements derived from *B*. *malayi* (15,412), *O*. *volvulus* (1,016), *W*. *bancrofti* (872) and *Wolbachia* (*wBm*, 804 genomic elements) genomic information. This microarray was used in many studies to analyze expression profiles during development and after drug treatments [36–42]. With new sequencing technologies, RNAseq has now become the more common tool to study stage-specific expression profiles of filarial worms [43,44] and the effects of known drugs on the worm’s transcriptome [45–47].

In 2008, the secretome of adult *B*. *malayi* worms was profiled from the proteomic characterization of the excretory-secretory (ES) products. The goal was to identify proteins that potentially influence infection by down-modulating host immune responses. Interestingly, among the more prominent novel products identified in the ES were a set of 11 small transthyretin-like proteins, which have been identified as potential vaccine candidates against other human helminth infections [48]. This analysis also identified novel proteins not previously suspected to be involved at the host-parasite interface, and thus provided important new insight on the biology of the filarial parasite [49]. The next year, a large-scale proteomic analysis also characterized the ES products of other stages such as L3, L3 to L4 molting worms, and microfilariae. Importantly, this analysis confirmed the presence of 274 "hypothetical" ES proteins inferred from gene prediction algorithms applied to the *B*. *malayi* genome. Moreover, it verified the enrichment of the previously characterized immunomodulatory proteins such as ES-62, leucyl aminopeptidase, MIF-1, serpin, glutathione peroxidase, and galectin in the ES of microfilariae and fertile adult females as compared to the adult males. It also revealed that many *Wolbachia*-specific proteins, most of which are metabolic enzymes, were released in the ES. These analyses expanded our knowledge of secreted proteins that could play a role in host-parasite interactions [50,51].

In 2011, another large-scale proteomic characterization of almost all the major mammalian stages of *B*. *malayi* was performed, resulting in the identification of more than 61% of the products predicted from the *B*. *malayi* draft genome as well as 63% of the *Wolbachia* proteome [52,53]. Analysis of protein families and domains coupled with stage-specific gene expression from microarray and RNASeq data highlighted the important pathways that benefit the parasite during its development in the host. Gene set enrichment analysis identified extracellular matrix proteins and those with immunologic effects as enriched in the microfilariae and L3 stages. Sex- and stage-specific protein expression identified those pathways related to parasite differentiation and demonstrated stage-specific protein expression by the *B*. *malayi* endosymbiont *Wolbachia* as well. Like most nematodes, filarial parasites have a fully formed digestive tract; however, its functionality was not completely clear. The tissue-specific proteomic analysis of the body wall, digestive tract, and reproductive tract of *B*. *malayi* clearly indicated enrichment in transporters within the digestive tract, suggesting that the intestine of adult filarial parasites is functional and important for nutrient uptake or waste removal. In addition, it revealed the presence of 27 possible vaccine candidates sequestered within the digestive tract with a high degree of homology to *W*. *bancrofti* or *O*. *volvulus*; these could possibly represent "hidden antigens" with low risk of prior allergic sensitization [54].

In 2016, the first high-quality genome assemblies with reconstruction of whole chromosomes for both *O*. *volvulus* [55] and *B*. *malayi* (WormBase.org) [Manuscript in preparation] were obtained. The transcriptomic and proteomic profiles of both *O*. *volvulus* and its *Wolbachia* endosymbiont (*w*Ov) in the major vector and the human host stages (L1, L2, L3, molting L3, L4, adult male and adult female) were also recently described [56]. This allowed the identification of stage-specific pathways important to the parasite’s adaptation to its human host during its early development.

**Fig 1:**
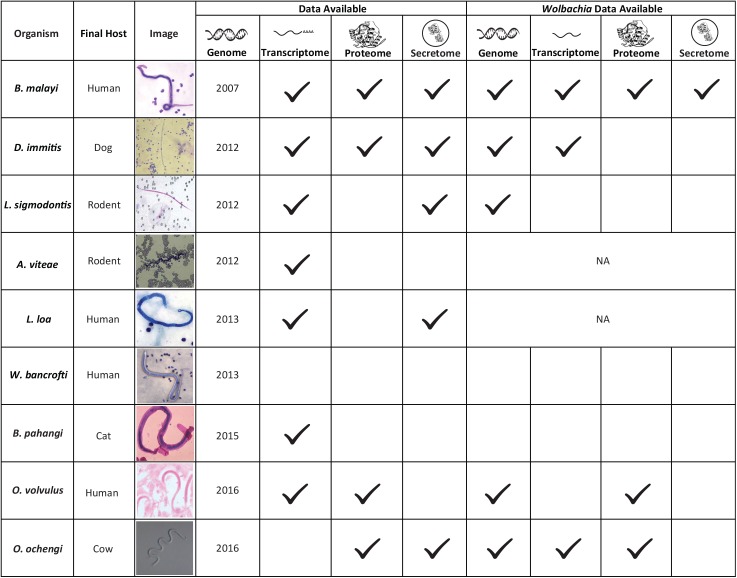
Summary of ‘omics data available for filarial parasites. Genomic, transcriptomic, proteomic and secretomic datasets of human and animal filarial parasites presently accessible for analyses.

Since 2010, a number of other draft genomes, transcriptomes and/or proteomes have also been published for other filarial nematodes including *L*. *loa*, *W*. *bancrofti*, *B*. *pahangi*, *D*. *immitis*, and *O*. *ochengi* [36,57–63] (Fig 1). The genomes of *Litomosoides sigmodontis and Acanthocheilonema viteae* are also available and can be found on the website http://nematodes.org/genomes/index_filaria.html.

It is clear that the recent advances in the sequencing of filarial genomes, transcriptomes and proteomes, as well as those of their bacterial endosymbionts, have contributed greatly to a better understanding of the biology of these parasites. A recent review [64] focuses on many pathways that have just come to light as being important for the establishment of the filarial parasites in their definite host as well as those that might contribute to the intricate host-parasite interactions in each parasite system. Examples include the discovery of novel immunomodulators, and a family of proteases and protease inhibitors essential for development and tissue migration that are also able to manipulate the immune system of their host. The analysis also points to the potential stage-specific provisioning of metabolic products by *Wolbachia* to the filarial worms. The hope is that a better understanding of the specific mechanisms that define the mutualistic interplay between the filarial parasites and their *Wolbachia* endosymbiont will facilitate the identification of pathways that could be targeted to ultimately kill the adult worms. Such novel macrofilaricidal drugs would complement present MDA efforts to control and eventually eliminate filariasis.

The next sections focus on how recent genomic advances for the filariae have also enabled the identification of novel drug targets, potential vaccine candidates and additional diagnostic biomarkers of *O*. *volvulus* infection. These represent three additional tools that will eventually support elimination of filarial infections.

## Drug repurposing and chemogenomic screening

As current microfilaricidal drugs appear to be insufficient for the control and elimination of these parasitic infections, new drugs will be required. Present efforts focus on the screening of libraries of drugs, including FDA repurposed drugs, against adult *Brugia* and *Onchocerca* worms *in vitro* and the selection of those that are effective for additional pre-clinical development and testing in small animal models [65–67]. While the primary focus is development of a macrofilaricidal drug candidate for the treatment of onchocerciasis, it is expected that parallel screening of the closely related filarid, *Brugia*, will also yield drug candidates for the treatment of lymphatic filariasis. An example is the discovery of auranofin as a potent anti-filarial drug [68]. Auranofin is an FDA-approved gold compound (2,3,4,6-tetra-O-acetyl-1-thio-beta-D-glucopyranosato-S (triethylphosphine) gold) that has been used to treat rheumatoid arthritis for over 25 years. In the study described by Bulman *et al*. [68] a library of over 2,000 FDA-approved compounds was screened first on *B*. *malayi* adult female worms and only auranofin was highly effective in inhibiting adult *Brugia* motility. It was then also shown to inhibit molting of *O*. *volvulus* and to kill adult *O*. *ochengi* worms. Additional studies will need to be conducted to determine efficacy with short treatment regimens *in vivo* using animal models and to obtain pharmacokinetic data before moving on to clinical development. Another approach is to screen repurposed and approved drugs from the human pharmacopoeia. This can be most easily done to target the *Wolbachia* endosymbionts of filarial worms, since macrofilaricidal effects have been observed when there is at least 90% reduction in the bacterial load [69,70], achievable with certain antibiotics.

Chemogenomic screening is a novel approach that uses a “chokepoint” analysis to identify essential drug targets. Reactions are determined to be essential if they either consume a unique substrate or produce a unique product. Based on the study reported by Taylor *et al* [71], a comparative analysis of nematode genomes yielded 487 genes conserved among all nematode species studied, of which 169 encoded chokepoint enzymes. A similar comparative analysis of the nematode proteomes yielded 477 chokepoint enzymes; 24 of which were found only in parasitic worms. Notably, several drugs that are already known anthelmintic drugs and novel candidate targets were identified in this study, 7 of which were tested in *Caenorhabditis elegans* and 3 that led to a detrimental phenotype. One of these three drug-like compounds, Perhexiline, was also deleterious to two parasitic nematodes, *Haemonchus contortus* and *O*. *lienalis* that exhibit different forms of parasitism and tropism in their final host. This study clearly illustrates that testing experimentally compounds already available and known to target proteins orthologous to nematode chokepoint proteins may lead to the identification of novel anthelminthics.

Filarial genomic, transcriptomic and proteomic data were also used in a computational target-based approach to screen FDA-approved drugs across all World Health Organization Anatomical Therapeutic Classes (WHO ATC) [55,63]. Sixteen *O*. *volvulus* enzymes and proteins involved in ion transport and neurotransmission were found to likely be good drug targets [55]. As some of these proteins are the targets of already approved human drugs that have not yet been tested on the filarial parasites, it would be of interest to verify if they could be repurposed as new therapies for filarial infections. Another computational approach, Flux Balance Analysis, calculates the flow of metabolites through metabolic networks constructed based on available enzyme annotation data to better understand the metabolic potential of a pathogen. This method was used to reconstruct the metabolism of two filarial nematodes, *O*. *volvulus* and *L*. *loa*, comparing essential reactions with specific interest in identifying those contributed by *Wolbachia* in the case of *O*. *volvulus* as *L*. *loa* does not harbor *Wolbachia*. Such analyses revealed that *O*. *volvulus* likely benefits from *Wolbachia* contributions to fatty acid metabolism, heme synthesis, and purine and pyrimidine metabolism. This analysis also pointed to the possibility that with the same gene complement, *O*. *volvulus* and *L*. *loa* may do things differently as it relates to certain pathways. For example, in purine salvage, *Wolbachia* provides an alternate pathway for *O*. *volvulus*, whereas *L*. *loa* depends exclusively on adenine import. This demonstrates how the presence of *Wolbachia* can change the metabolic chokepoints in filaria and serves to identify selective drug targets [55].

## High-throughput immunomic screening of *O*. *volvulus* vaccine candidates

There are two types of vaccines that would be necessary for the efficient control of onchocerciasis: (a) a *prophylactic* vaccine to be used in children <5 years old to block new infections and the accumulation of adult worms, thus reducing microfilarial densities in the skin, pathology and transmission; and (b) a *therapeutic* vaccine that would be used in older children and adults that already carry adult worms, to potentially impair the fertility of female parasites, suppress the release of nodular microfilariae from the female worm and/or kill them once released, reducing accumulation of skin microfilariae thus interrupting the transmission cycle [72]. In both cases, the recipient of the vaccine benefits from a reduction in the only *O*. *volvulus* parasite stage that causes disease, the microfilaria. Importantly, the entire community also benefits since the microfilaria is the transmissible stage to insect vectors, further protecting areas from recurrence transmission where local elimination may have already been achieved. Vaccines may also lower the number of annual MDA with ivermectin, forestalling drug resistance, and ensuring the success of the existing MDA. Most of the present *O*. *volvulus* vaccine candidates were discovered by screening expression libraries with various antibody probes [73]. Two *O*. *volvulus* vaccine proteins, *Ov*-103 and *Ov*-RAL-2, are promising candidates for prophylactic vaccine [74] and their homologues in *B*. *malayi* were shown to also induce protection against infection with *B*. *malayi* infective stage larvae [75]. The disappointing results of clinical trials for several infectious diseases highlight the current limitations of vaccine candidate selection approaches that often fail to exclude at an early stage antigens with poor immunogenicity or low safety profiles in humans [76,77]. One approach for identifying novel vaccine candidates is immunomics [78–82], which allows high-throughput profiling of the host immune antibody responses to genome-wide candidate parasite antigens. Using this approach with putatively immune human sera and sera from infected individuals [56], six new potential vaccine antigens were identified by screening antibody responses (IgG1, IgG3 and IgE) against an *O*. *volvulus* recombinant protein array containing 362 proteins. Four of these antigens are highly expressed during the early stages of larval development in the human host and thus could be tested for efficacy in a prophylactic vaccine. The 2 other proteins are highly expressed by the microfilariae and are specifically recognized by sera from protected individuals who never developed a patent infection. This opens new possibilities for developing a safe anti-transmission or therapeutic vaccine. To the best of our knowledge, this is the first and only occasion in which genome-wide stage-specific expression data from *O*. *volvulus* have been exploited to discover novel vaccine candidates in an unbiased manner. Future studies using the diffusion chamber mouse model for *O*. *volvulus* will confirm whether these antigens do indeed protect against infection by L3s or against microfilariae [83].

## Identifying novel *O*. *volvulus* biomarkers of infection

Gold standard diagnosis using blood films or skin snips has become less relevant as mass drug distribution programs for control of filarial infections have expanded. The spectrum of programmatic processes (mapping, mass drug interventions, monitoring and evaluation, and surveillance) require different approaches as different questions are asked at each stage [84]. Infection intensity may refer to adult worm burden or microfilarial load in the skin of *O*. *volvulus* infected individuals. However, the relationship between microfilarial load, as assessed by quantification of microfilaridermia by skin snip in onchocerciasis, and the total adult parasite burden is at best semi-quantitative. Moreover, the current toolbox for diagnosis and surveillance of onchocerciasis, as well as other helminthic infections, is limited because many of the available tools suffer from lack of sensitivity and specificity and/or are cost-prohibitive [85,86]. Given the constraints of achieving elimination using MDA with ivermectin alone, and concerns about recrudescence in areas of previous onchocerciasis control, more efficient tools are needed for diagnosis and monitoring of current and future control measures using emerging technologies that are field-deployable or suitable for low-resource settings. The development of better diagnostic tools is greatly needed for post-treatment surveillance where transmission of infection has been brought under control, the certification phase, and for mapping prevalence in meso- and hypo-endemic areas that had heretofore been ivermectin-naive. Transcriptome and proteome data have helped in the discovery of new biomarkers for *O*. *volvulus* infection. Using immunomics and the *O*. *volvulus* protein array used for the discovery of vaccine candidates, we identified 7 previously unrecognized biomarkers of active patent infection (OVOC10469, OVOC10602, OVOC11950, OVOC3261, OVOC5127, OVOC8491, OVOC9988), based on IgG4 responses in infected individuals [56]. Future assays, such as a luciferase immunoprecipitation system (LIPS) immunoassay, will help validate if such highly antigenic *O*. *volvulus* proteins can be used as specific and sensitive biomarkers of patent infection.

In another recent study [87], an integrated approach was used to identify adult female *O*. *volvulus* antigens to be explored for developing serodiagnostic tests. Using data from the *O*. *volvulus* genome, proteome, and transcriptome, 241 immunoreactive proteins were identified. These included most of the major diagnostic antigens described over the past 25 years, validating the approach, plus 33 new proteins with great promise as serodiagnostic antigens. These candidates, as well as those identified using the immunomics approach together with the extensive pan-omics dataset generated in the studies described above, will facilitate the development of novel diagnostic tools. When combined with present and future secretome datasets, there is hope that additional tests focused on antigen detection assays in body fluids could also be developed.

## Conclusion

The abundance of genomic, transcriptomic, and proteomic data has already provided novel biological insight into filarial nematodes, and led to the identification of novel drug targets, vaccine candidates and biomarkers of infection. We should expect that upcoming exploitation of these various novel datasets will further our understanding of these unique parasites and their interaction with the final host, ultimately helping us reach the goal envisioned by WHO to eliminate filarial infections for good [88].

**Key learning points for the role of ‘omics’ in the quest to eliminate human filariasis**

- Although there has been much progress in the research and control of human filariasis, major obstacles remain that challenge the global public health community and for which fundamental and applied research is urgently needed.
- Genomes of filarial parasites are becoming increasingly available and through the related advances in transcriptomics and proteomics promise to revolutionize the field of helminth filarial biology and help unravel new targets for control and novel diagnostic tools.
- Without accurate annotation and the development of novel functional genomic tools, these data will not be truly valuable to support the better understanding of the filarial biology, host-parasite interactions and symbiosis.
- Knowledge of factors controlling host–parasite interactions can ultimately support identification of vulnerable pathways to be targeted by novel interventions and help avert unintended consequences of intervention (e.g., increased transmission, and/or morbidity).
- Knowledge of how parasite population genetic structure will change under chemotherapeutic pressure is essential to understand the evolutionary implications of intervention.
- The processes that initiate and sustain immune regulation on the one hand, or lead to pathogenesis on the other and the effects upon them of prolonged anthelmintic intervention remain incompletely understood.
- Twenty four publications cited in this review were disseminated through publication in PLOS NTD.

**Key papers in the field of ‘omics’ in filariasis**

- Fischer PU, King CL, Jacobson JA, Weil GJ (2017) Potential Value of Triple Drug Therapy with Ivermectin, Diethylcarbamazine, and Albendazole (IDA) to Accelerate Elimination of Lymphatic Filariasis and Onchocerciasis in Africa. PLoS Negl Trop Dis 11: e0005163.
- Kim YE, Remme JH, Steinmann P, Stolk WA, Roungou JB, et al. (2015) Control, elimination, and eradication of river blindness: scenarios, timelines, and ivermectin treatment needs in Africa. PLoS Negl Trop Dis 9: e0003664.
- Foster J, Ganatra M, Kamal I, Ware J, Makarova K, et al. (2005) The Wolbachia genome of Brugia malayi: endosymbiont evolution within a human pathogenic nematode. PLoS Biol 3: e121.
- Ghedin E, Wang S, Spiro D, Caler E, Zhao Q, et al. (2007) Draft genome of the filarial nematode parasite Brugia malayi. Science 317: 1756–1760.
- Desjardins CA, Cerqueira GC, Goldberg JM, Dunning Hotopp JC, Haas BJ, et al. (2013) Genomics of Loa loa, a Wolbachia-free filarial parasite of humans. Nat Genet 45: 495–500.
- Cotton JA, Bennuru S, Grote A, Harsha B, Tracey A, et al. (2016) The genome of Onchocerca volvulus, agent of river blindness. Nature Microbiology 2: 16216.
- Bennuru S, Cotton JA, Ribeiro JM, Grote A, Harsha B, et al. (2016) Stage-Specific Transcriptome and Proteome Analyses of the Filarial Parasite Onchocerca volvulus and Its Wolbachia Endosymbiont. MBio 7.
